# Bovine Viral Diarrhea/Mucosal Disease—A Commentary of the Guest Editor

**DOI:** 10.3390/vaccines10040590

**Published:** 2022-04-12

**Authors:** Kerstin Wernike

**Affiliations:** Institute of Diagnostic Virology, Friedrich-Loeffler-Institut, 17493 Greifswald-Insel Riems, Germany; kerstin.wernike@fli.de

Bovine viral diarrhea (BVD) is one of the most significant cattle diseases worldwide, and control programs have been implemented in several countries. The causative agent, bovine viral diarrhea virus (BVDV), is a flavivirus that exists in the two distinct species *Pestivirus A* (BVDV type 1) and *Pestivirus B* (BVDV type 2). Vaccination can locally protect herds from virus introduction and could assist in BVDV eradication programs [1].

The main source for the spread and perpetuation of BVDV are in utero infected, immunotolerant, persistently viremic calves, which shed enormous amounts of virus throughout their lives, since they are incapable of mounting a specific immune response against the virus strain they are infected with. Hence, fetal protection against all circulating BVDV strains is a major benchmark of vaccination strategies. Considering the pivotal role of fetal infections, Arnoux and colleagues assessed in their within-herd epidemiological model the economic impact of BVD and the reward associated with the vaccination of breeding females for two possible protection scenarios: (1) protection of the dam against infection or (2) protection against vertical transmission from the dam to the fetus [2]. In addition, the specificities of three distinct cow-calf farming systems as well as the external risk of virus introduction and the characteristics of diverse commercial vaccines were taken into account. In the breeding system named “Charolaise”, the economic impact over 5 years after BVDV introduction was EUR 113 per cow. When all breeding females were vaccinated yearly and when the external risk for virus introduction was high enough, the yearly expected reward was EUR 0.80 per invested euro per cow, irrespective of the vaccine. However, in the two other analyzed breeding systems, the median reward was almost nil [2]. Interestingly, of the three farming systems, the grazing period is the longest in the “Charolaise” system, leading to longer periods of exposure to animals from neighboring herds [2]. Contacts with other herds on pasture and the purchases of potentially infected animals may lead to a higher risk of virus reintroductions. Hence, the herd characteristics and the epidemiological situation influence the BVD impact and should be considered when a decision for or against vaccination is to be made.

Another factor contributing to the efficiency of vaccination programs in addition to the farming system is the longevity of the antibody response in vaccinated cattle, which impacts on the required interval of re-vaccinations of individual animals. In this article collection, Klimowicz-Bodys et al. investigated the antibody response of dairy cows to a single dose of a commercial modified-live virus vaccine [3]. Blood samples collected for a year at monthly intervals were tested, and the antibody titers remained high over this period. Besides, no reproduction disorders were observed during the study period, and all newborn calves tested negative for BVDV antigen. Thus, the authors concluded that a single vaccination with the modified-live virus vaccine stimulated a strong antibody response lasting for at least 12 months and increased the herd immunity, thereby reducing the risk for acute infections and the percentage of persistently infected calves when applied in combination with a comprehensive BVDV control program [3].

In both aforementioned studies, well-established, commercially available vaccines were evaluated or included in the modelling; however, none of them enable a serological differentiation of infected from vaccinated animals (DIVA strategy). A marker vaccine could be beneficial in the monitoring of vaccine efficiencies in endemically infected regions with ongoing control programs. Furthermore, it permits the demonstration of freedom from the disease by serological methods when applied in the context of emergency vaccinations during a BVDV outbreak in non-endemic countries. Therefore, the DIVA capability is an important point to bear in mind when developing novel anti-BVDV vaccines.

In this Special Issue, two different approaches are described to express the immunodominant proteins of BVDV. While Wang et al. selected a baculovirus expression vector system to generate BVDV virus-like particles (VLP) [4], Köthe et al. made use of the interchangeability of individual genes between different *Pestivirus* species [5], which is permitted by the high similarity in the genome organization of the members of this virus group. Here, a BVDV type 1 double marker vaccine was constructed based on the genetic background of the atypical pestivirus Bungowannah virus (BuPV, species *Pestivirus F*) expressing the heterologous glycoproteins E1 and E2 of BVDV type 1. The candidate vaccine was attenuated by the introduction of a deletion within the coding sequence of the N^pro^ protein, which is a major type I interferon inhibitor. Immunization of cattle, the target species of BVDV, with the chimeric virus either as a modified-live virus vaccine applied once or as inactivated preparation injected twice, followed by a BVDV challenge infection, confirmed the safety of the candidate vaccine and demonstrated the induction of a robust immune response and a high level of clinical protection [5]. The serological discrimination of vaccinated cattle was enabled by the detection of antibodies against the E2 protein of BVDV in the absence of both BVDV NS3- and E^rns^-specific antibodies [5].

For the VLPs that are described to be non-infectious and genome-free particles composed of the viral proteins E2 and E^rns^ [4], a future DIVA approach might be based on the presence of antibodies against these proteins in the absence of antibodies directed against the non-structural protein NS3 of BVDV. In the VLPs, E2 and E^rns^ existed in homodimers, which could help to preserve the immunogenicity of the two proteins. To investigate the humoral and cellular immune responses subsequent to vaccination in an in vivo model, Wang et al. immunized mice with the BVDV-VLPs. While the VLPs alone induced low levels of IgG, IgG1 and IgG2a, immunization with VLPs combined with an adjuvant resulted in a strong induction of the immune response, comparable to that induced by immunization with a commercial inactivated vaccine formulation [4]. Therefore, the BVDV-VLPs generated using a baculovirus expression vector system showed the promising potential of VLPs for the development of vaccines against BVDV infections [4].

Taken together, this collection of article highlights some advances in the development of vaccine platforms and vaccination strategies against BVDV.
